# 

**Published:** 1883-02

**Authors:** 


					﻿NOTICE.
All articles for publication, books for review, business communications, etc.,
must be sent to Editor, 2109 Chestnut fitreet, Philadelphia.
CONTRIBUTORS:
Drs. H. C. Allen, T. F. Allen, Edward Bayard, J. B, Bell, E. W. Berridge,
Geo. H. Clark,A. C- Cpwperth waite, Win. J. Guernsey, W. A. Hawley,
John Hall,- W. M. James, W. H. Jenny, Ad. Lippe, C. Lippe,
Malcolm MacFarlan, C. F. Nichols, C. Pearson, T. F. Pom-
eroy, Leila A. BenDell, C. Carleton Smith, P. P. Wells,
Wm. P. Wesselhoeft, David Wilson (London),
T. P. Wilson, and many others.
Authors of accepted papers are furnished with copies of the
number in Which their articles appear^ application for such copies
to ;be made when sending papersi Any irregularity in the receipt
of this Journal should bp promptly reported, to Editor.
				

